# Fibrinogen decreases cardiomyocyte contractility through an ICAM-1-dependent mechanism

**DOI:** 10.1186/cc6213

**Published:** 2008-01-03

**Authors:** John H Boyd, Edmond H Chau, Chiho Tokunanga, Ryon M Bateman, Greg Haljan, Ehsan Y Davani, Yinjin Wang, Keith R Walley

**Affiliations:** 1 University of British Columbia Critical Care Research Laboratories, St. Paul's Hospital, 1081 Burrard Street, Vancouver, BC, V6Z 1Y6, Canada

## Abstract

**Introduction:**

Cardiomyocytes exposed to inflammatory processes express intracellular adhesion molecule-1 (ICAM-1). We investigated whether fibrinogen and fibrinogen degradation products, including D-dimer, could alter cardiomyocyte contractile function through interaction with ICAM-1 found on inflamed cardiomyocytes.

**Methods:**

*In vivo*, rats were injected with endotoxin to model systemic inflammation, whereas isolated rat cardiomyocytes were treated with tumor necrosis factor-alpha to model the inflammatory environment seen following exposure to bacterial products such as lipopolysaccharide.

**Results:**

*In vivo*, endotoxin administration profoundly decreased cardiac contractile function associated with a large increase in intracardiac ICAM-1 and perivascular fibrinogen. Confocal microscopy with double-staining of isolated rat cardiomyocytes demonstrated colocalization of ICAM-1 and fibrinogen. This interaction was disrupted through pre-treatment of the cells with an ICAM-1-blocking antibody. Functionally, isolated rat cardiomyocyte preparations exhibited decreased fractional shortening when incubated with fibrinogen, and through the use of synthetic peptides, we determined that residues 117–133 of the fibrinogen gamma chain are responsible for this interaction with ICAM-1. Despite having crosslinked gamma chains, D-dimer retained the ability to decrease cardiomyocyte contractility.

**Conclusion:**

Site 117–133 of the fibrinogen gamma chain is able to depress cardiomyocyte contractility through binding ICAM-1.

## Introduction

Both local and systemic inflammation impair cardiac contractility, although the precise mechanism behind this is still unclear [1-3]. It is now recognized that high levels of inflammatory biomarkers such as C-reactive protein and D-dimer are associated with an increased incidence of, and worse prognosis for, cardiovascular disease [4-8]. However, whether these molecules are simply markers of the inflammatory process or might actually play a causative role in the resultant organ dysfunction is not known. We previously reported a novel two-step regulatory mechanism of cardiomyocyte contractility whereby systemic inflammation induces cardiomyocyte-expressed intracellular adhesion molecule-1 (ICAM-1), whose subsequent ligation results in decreased contractility by signaling via the cytoskeleton [9]. While studies using cardiomyocyte/leukocyte co-culturing methods demonstrate that activated leukocytes can bind to ICAM-1 with a resultant decrease in myocardial contractility [9-11], we and others have noted a paucity of intramyocardial leukocytes in whole animal models of inflammation [12,13]. Therefore, we postulated that in the more complex environment of an *in vivo *model, ICAM-1 ligands other than the CD11/CD18 receptors found on activated leukocytes [14-16] play a greater role in ICAM-1-dependent decreases in cardiomyocyte contractility.

Fibrinogen, a 340-kDa plasma glycoprotein with a physiological plasma concentration of 1.5 to 4.5 g/L, as well as its related protein fragments D-dimer and other fibrinogen degradation products (FDPs) represent potential ICAM-1-binding myocardial depressant substances. The amino acid sequence 117–133 of fibrinogen gamma chain (fg-γ-117–133) binds to the amino acid sequence 8–22 (ICAM-1-8–22) within the first immunoglobulin (Ig) domain of ICAM-1 [17]. The functional role of the fibrinogen-ICAM-1 interaction includes adhesion of leukocytes to endothelial cells [18,19], leukocyte transmigration [20], and promotion of endothelial cell survival [21]. Interestingly, fibrinogen-ICAM-1 ligation leads to cytoskeleton-dependent ERK1/2 (extracellular signal-regulated kinase-1/2) phosphorylation in endothelial cells [22]. In view of our previous observation in cardiomyocytes that ICAM-1 ligation by leukocytes reduces contractility via focal adhesion kinase phosphorylation at the cytoskeleton [9], fibrinogen-ICAM-1 ligation ultimately could lead to alteration in cardiomyocyte contractile function. As fragments of polymerized fibrinogen such as D-dimer are markedly elevated in most inflammatory states [4-8], it is of particular interest to determine whether these molecules are able to influence cardiac physiology through interaction with ICAM-1.

The goal of our study, therefore, was to determine whether exposure to fibrinogen and FDPs altered the contractile function of cardiomyocytes. To simulate systemic inflammation, rats were injected with endotoxin, and through immunohistochemistry, we confirmed an increase in both cardiomyocyte-expressed ICAM-1 as well as increased intramyocardial fibrinogen deposition. In isolated cardiomyocytes exposed to an inflammatory environment, we established the specificity of the fibrinogen-ICAM-1 interaction and went on to determine the active site on fibrinogen responsible for ICAM-1-mediated alterations in contractility.

## Materials and methods

This study was approved by the University of British Columbia Animal Care Committee and adheres to the Canadian and National Institutes of Health guidelines for animal experimentation.

### *In vivo *experimental models

For the endotoxin model of inflammation, male Sprague-Dawley rats 350 to 450 g in weight were injected intraperitoneally with lipopolysaccharide (LPS) (10 mg/kg) or vehicle control (normal saline). The LPS dosage was selected as a midrange dosage of endotoxemic models that result in a hemodynamic effect [23,24]. The heart was excised 6 hours after injection, embedded in Optimal Cutting Temperature compound (Electron Microscopy Sciences, Hatfield, PA, USA), frozen in dry-ice-chilled isopentane, and stored at -80°C.

### Measurement of left ventricular contractility and cardiac function

Left ventricular contractility and other measures of ventricular function were determined from pressure-volume measurements using Pressure-Volume Analysis software (PVAN 2.9; Millar Instruments Inc., Houston, TX, USA). Six to ten pressure-volume loops during a vena cava occlusion were sampled and used to measure end-systolic elastance (E_es_), which is the slope of the end-systolic pressure-volume relationship relatively insensitive to changes in preload and afterload [25]. E_max _is defined as the maximal E_es_. The volume axis intercept, Vd, was considered zero volume for the steady-state measurements. Pressure-volume loops measured during steady-state conditions were used to measure the maximum rate of change of intraventricular pressure during isovolumic systole divided by end diastolic volume (EDV), (dP/dt_max_)/EDV, which is a sensitive isovolumic phase measure of left ventricular contractility [26], as well as to calculate ejection fraction (EF). End-systolic pressure during steady state was used as a measure of systemic arterial pressure afterload.

### Immunofluorescent imaging with quantification

Frozen heart sections (6 μm) were acetone-fixed and incubated with universal blocking agent (DakoCytomation, Glostrup, Denmark). Fibrinogen and von Willebrand factor (vWF), a marker for the endothelium, were stained together to assess whether infiltration of fibrinogen into the myocardium occurred. Sections were incubated with 1:20 fluorescein isothiocyanate (FITC)-conjugated goat anti-mouse fibrinogen (Nordic Immunological Laboratories, Tilburg, The Netherlands) and 1:200 rabbit anti-mouse vWF (DakoCytomation) primary antibody and then labeled with Alexa Fluor 594 goat anti-rabbit antibody (Invitrogen Corporation, Carlsbad, CA, USA). Nuclei were stained with the Hoechst stain (Invitrogen Corporation). Control sections were incubated with FITC-conjugated non-specific goat IgG (Santa Cruz Biotechnology, Inc., Santa Cruz, CA, USA) and non-specific rabbit IgG (DakoCytomation) and processed in identical conditions.

Immunofluorescent ICAM-1 staining was carried out by incubating sections with 1:500 mouse anti-rat ICAM-1 monoclonal antibody 1A29 (BD Biosciences, San Jose, CA, USA) and then with Alexa Fluor 594-labeled goat anti-mouse antibody (Invitrogen Corporation). Control sections were incubated with non-specific mouse IgG (Invitrogen Corporation). After drying, the slides were mounted with DABCO (1,4-diazabicyclo[2.2.2]octane) to prevent photobleaching.

Images were captured using a laser scanning confocal microscope with a 63× water immersion lens (Leica SP2; Leica, Wetzler, Germany). Samples were imaged using fluorescence with wavelength excitations and emissions of 488 nm and 495 to 580 nm (respectively) for fibrinogen and 594 nm and 600 to 700 nm (respectively) for vWF and ICAM-1. The scan format was 512 × 512 pixels. Image capturing was performed sequentially using a three-frame average. All imaging was performed under identical microscope settings (for example, laser intensity and photomultiplier tube gain).

Cross-sections of 15 randomly selected blood vessels, identified via vWF staining, were imaged. Two ellipses were traced around each vessel: a small ellipse positioned closely along the vessel boundary and a large ellipse with proportional major and minor axes but three times the area of the small ellipse. Fibrinogen staining present in the annulus between the two ellipses was identified as perivascular fibrinogen. The sum fluorescence intensity per annulus area was measured using the Leica software.

To measure myocardial ICAM-1 expression, heart sections from the endotoxemic and control groups were imaged as described above and fluorescent intensity measures were taken using traced field areas containing myocardial tissue. The sum fluorescence intensity per unit area was measured using the Leica software.

### Isolation of rat ventricular myocytes

Male Sprague-Dawley rats were injected with heparin and anesthetized using isofluorane. The heart was excised, mounted on a modified Langendorff apparatus, and digested with 281 U/mL collagenase (Worthington Biochemical Corporation, Lakewood, NJ, USA). After digestion, the cells were resuspended in modified Eagle's medium containing increasing Ca^2+ ^concentrations (200 μM, 500 μM, and 1 mM). Five hundred thousand cells in M199 with bovine serum albumin (BSA) were loaded into a laminin-coated Petri dish 6 cm in diameter (BD Biosciences) and the cardiomyocytes were incubated for 12 hours to allow them to become relatively quiescent. After 24 hours, cells were considered viable if they demonstrated a characteristic rod shape without cytoplasmic blebbing.

### Measurement of cardiomyocyte fractional shortening

Cells were paced at 1 Hz using a Grass S48 stimulator (Grass-Telefactor, Warwick, RI, USA) with a voltage set at 120% of the threshold capture voltage. Images were captured using a Myocam video camera (IonOptix Corporation, Milton, MA, USA) and analyzed using an IonOptix Softedge detection package (IonOptix Corporation). Fractional shortening was calculated as the difference between diastolic and systolic lengths, divided by diastolic length.

### Coating of fibrinogen to polystyrene beads

Polystyrene beads 8 μm in diameter (Bangs Laboratories, Inc., Fishers, IN, USA) were washed twice with acetate buffer (pH 5.4). Beads were mixed with rat fibrinogen (Enzyme Research Laboratories, South Bend, IN, USA) at a concentration of 300,000 beads per microgram of fibrinogen in a 500-μL Eppendorf tube. A micromagnetic stir bar was placed in the tube, and the mixture was gently stirred for 2 hours at room temperature. The beads were then washed three times with fresh acetate buffer. Clumps of fibrinogen-coated beads were broken apart by passing them through a syringe with a 27.5-guage needle.

### ICAM-1 peptide-fibrinogen binding assay

Ninety-six-well Corning Costar 9018 enzyme-linked immunosorbent assay (ELISA) plates (eBioscience, Inc., San Diego, CA, USA) were coated for 2 hours at room temperature with fibrinogen concentrations ranging from 0.01 to 100 nM in bicarbonate/carbonate coating buffer (3.03 g of Na_2_CO_3_, 6.0 g of NaHCO_3 _per 1,000 mL of distilled water) (pH 9.6). Wells were washed with phosphate-buffered saline (PBS) and then blocked overnight with 1% BSA. One hundred micromolar biotinylated ICAM-1 (8–22) sequence EAFLPRGGSVQVNCS or biotinylated scrambled peptide sequence SCNVQVSGGRPLFAE (University of British Columbia Peptide Facility, Vancouver, BC, Canada) was then added to the wells and incubated for 2 hours at room temperature before washing three times with PBS. HRP-linked anti-biotin antibody (1 μg/mL) (Invitrogen Corporation) was then added and incubated for 2 hours, followed by three washes. One hundred microliters of ABTS (2,2'-azino-di(3-ethylbenzthiazoline sulfonate) solution (Chemicon International, Temecula, CA, USA) was added to each well and incubated for 60 minutes. Absorbance values were measured at 405 nm and at 492 nm for reference.

### Incubations

Twenty-four hours after cardiomyocyte isolation, cells were activated with tumor necrosis factor-alpha (TNF-α) (20 ng/mL) for 4 hours to upregulate ICAM-1 expression [9]. In studies using fibrinogen-coated beads, 25,000 beads were added to cells in laminin-coated 96-well plates (500 cells per well). A bead that moved with the contracting cardiomyocyte and maintained a contact relative location on the membrane during contraction was considered to be adherent. In studies using rat fibrinogen (Enzyme Research Laboratories), human fibrinogen, D-dimer fragments D and E (HYPHEN BioMed, Neuville-sur-Oise, France), and fibrinogen gamma chain (AnaSpec, Inc., San Jose, CA, USA) cells were incubated at concentrations of 0.03 μM, 0.1 μM, 0.3 μM, and 1 μM, respectively, for 4 hours at 37°C. After 4 hours at 37°C, cardiomyocyte fractional shortening was measured as described above.

In studies using the ICAM-1 (8–22) sequence EAFLPRGGSVQVNCS or scrambled peptide sequence SCNVQVSGGRPLFAE (University of British Columbia Peptide Facility), 1 μM rat fibrinogen and 100 μM ICAM-1 (8–22) or scrambled peptide were mixed and incubated for 4 hours at 37°C prior to incubation with cardiomyocytes as described above. In studies using anti-ICAM-1-blocking monoclonal antibody 1A29 (BD Biosciences), the antibody was added to cardiomyocytes at a concentration of 200 ng/mL 4 hours prior to the addition of fibrinogen.

### Colocalization of ICAM-1 with fibrinogen

Upon TNF-α activation, cardiomyocytes were co-cultured with Oregon Green-labeled fibrinogen (Invitrogen Corporation) for 4 hours. They were then fixed with 3% paraformaldehyde for 20 minutes and blocked with universal blocking agent. Immunofluorescent ICAM-1 staining was carried out by incubating the cardiomyocytes with 1:500 mouse anti-rat ICAM-1 antibody (BD Biosciences) followed by Alexa Fluor 594-labeled goat anti-mouse antibody. The cardiomyocytes were examined using a confocal microscope (Leica) as described in the previous section. The scan format was 1,024 × 1,024 pixels, and 2× zoom was applied.

### Statistical analysis

All data are expressed as mean ± standard error. For each experimental condition and time point, at least four independent replicate analyses were performed, unless otherwise noted. Differences between groups were tested using a one-way analysis of variance and the *post hoc *Bonferroni test to identify specific differences between groups. Differences were considered significant for *P *values of less than 0.05.

## Results

### Systemic inflammation depresses cardiac contractility and is associated with intracardiac extravasation of fibrinogen and increased expression of ICAM-1 by cardiomyocytes

**Figure 1:**
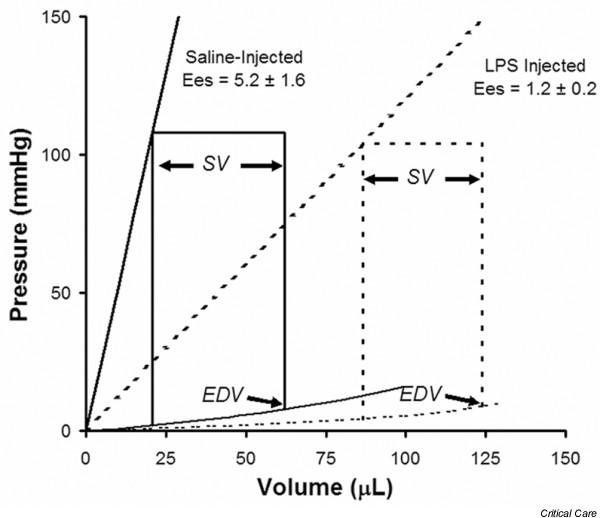
**LPS decreases cardiac contractility**. Cardiac cycle pressure-volume loops obtained 6 hours after intraperitoneal injection of lipopolysaccharide (LPS) or saline into rats. Acquired with group mean data, the curves demonstrate an LPS-induced increased end diastolic volume (EDV), maintenance of stroke volume (SV), and therefore a marked reduction of left ventricular ejection fraction (SV/EDV). E_es_, end-systolic elastance.

**Figure 2:**
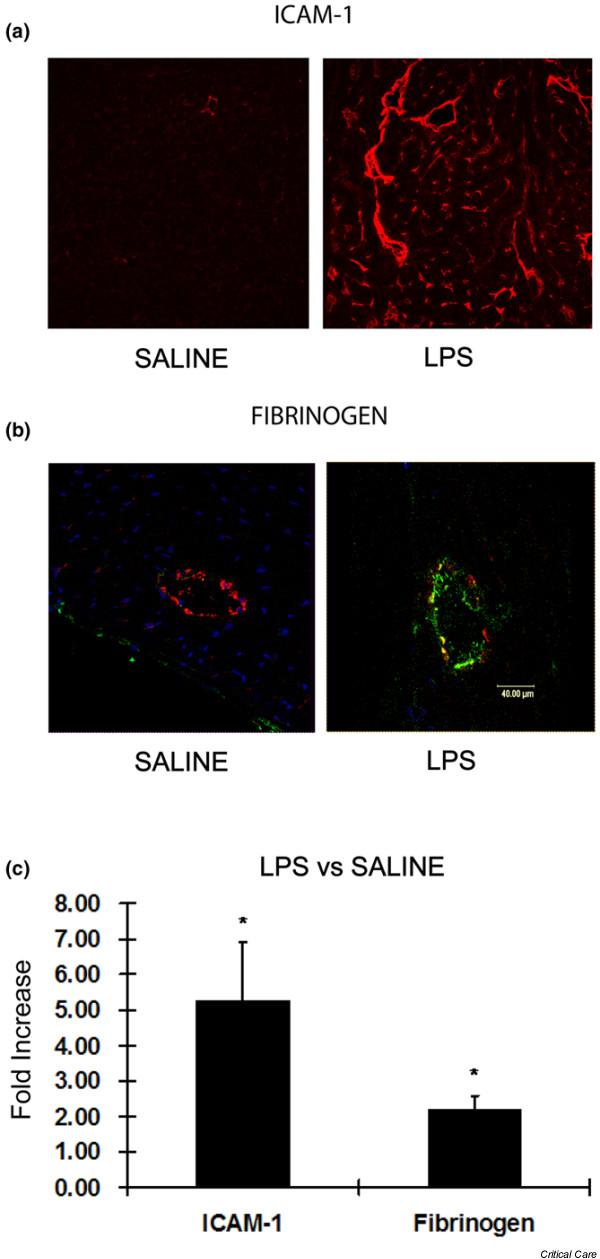
**LPS increases intracardiac ICAM-1 and perivascular fibrinogen**. **(a) **Frozen cardiac sections from lipopolysaccharide (LPS)-treated and saline-treated rats demonstrate that intracardiac intracellular adhesion molecule-1 (ICAM-1) (red) is dramatically increased in the former. **(b) **Fibrinogen (green) was greatly increased outside the endothelium (von Willebrand factor labeled red) in the LPS group compared with rats treated with saline. **(c) **Group mean data of the fold increases in myocardial ICAM-1 expression and perivascular fibrinogen deposition in LPS-treated versus saline-treated animals. *p < 0.05 versus saline.

We determined whether endotoxin would create an environment within the heart which would allow ICAM-1 expressed on cardiomyocytes to interact with extravasated fibrinogen and whether this would be associated with alterations in cardiac contractility. Six hours after LPS injection, the endotoxemic group of rats exhibited decreased left ventricular contractility compared with the saline-treated controls (Figure 1). Cardiac cycle pressure-volume loops using mean data clearly demonstrate an LPS-induced rightward shift along the volume axis. This shift reflects left ventricular dilation represented by increased EDV, maintenance of stroke volume (SV), and resultant marked reduction of left ventricular EF (EF = SV/EDV) (Figure 1 and Table 1). The preload-independent E_max _is dramatically decreased in LPS-treated versus saline-treated rats, whereas (dP/dT)/EDV reflects isovolemic contractility and is also depressed with LPS (Table 1). Immunostaining of heart tissue from these rats demonstrated a dramatic increase in both intramyocardial ICAM-1 expression and perivascular fibrinogen in the myocardium of LPS-treated rats (Figure 2a,b). Image quantification demonstrated a 5.5 ± 1.6-fold increase in ICAM-1 expression and a 2.1 ± 0.6-fold increase in perivascular fibrinogen in the myocardium of LPS-treated rats (Figure 2c).

**Table 1:** Hemodynamic data from endotoxemic and control animals

Treatment	Heart rate (beats per minute)	Systolic pressure (mm Hg)	Ejection fraction (percentage)	(dP/dT)/EDV	E_max_
LPS	392 ± 152	108 ± 8	38 ± 2	44 ± 3	2.5 ± 0.4
Control^a^	313 ± 30	112 ± 6	52 ± 8	121 ± 11	7.7 ± 0.6
	*P *= NS	*P *= NS	*P *< 0.05	*P *< 0.05	*P *< 0.05

^a^Saline-treated rats. dP, derivative of pressure; dT, derivative of time; EDV, end diastolic volume; E_max_, maximal end-systolic elastance; LPS, lipopolysaccharide-treated rats; NS, not significant.

### ICAM-1 expressed on activated isolated cardiomyocytes specifically binds to fibrinogen

**Figure 3:**
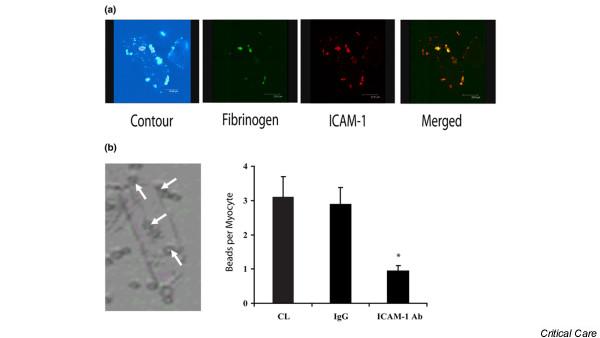
**Fibrinogen binds specifically to cardiomyocyte ICAM-1**. Colocalization of fibrinogen and cardiomyocyte intracellular adhesion molecule-1 (ICAM-1). **(a) **Isolated cardiomyocytes were incubated with Oregon Green-labeled fibrinogen (green) and fluorescently stained for ICAM-1 (red). A multiphoton dual-excitation image of the contour of the cell of interest is shown. By means of an overlay of images, strong colocalization of fibrinogen and ICAM-1 was indicated by a yellow color. **(b) **A representative image of a rat cardiomyocyte with adherent fibrinogen-coated polystyrene beads (white arrows) is shown to the left of the graph. The specificity of the ICAM-1-fibrinogen interaction is demonstrated as anti-ICAM-1 antibody pre-treatment results in significantly less fibrinogen-coated polystyrene beads adherent to the cardiomyocytes (*p < 0.05 versus control). Ab, antibody; CL, control; IgG, immunoglobulin G.

**Figure 4:**
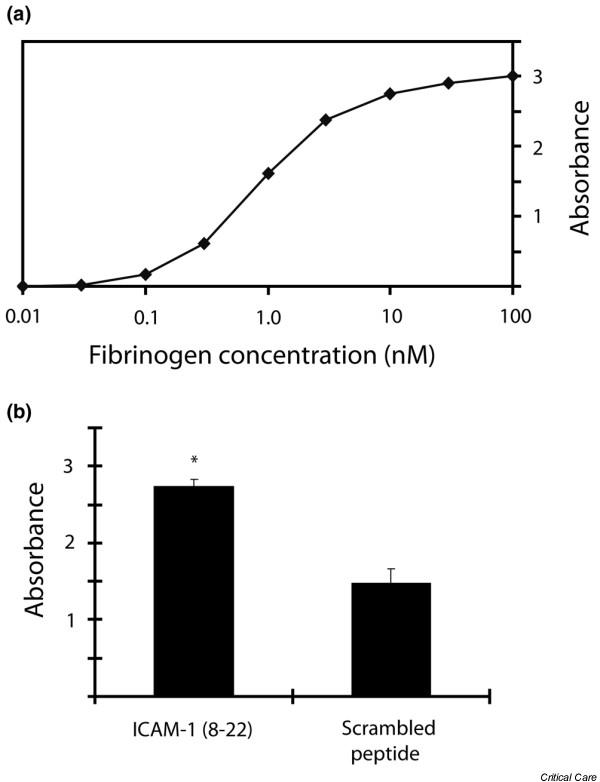
**ICAM-1 (8–22) binds to fibrinogen**. Two-step enzyme-linked immunosorbent assay in which fibrinogen was immobilized on the 96-well plate and then incubated with biotinylated intracellular adhesion molecule-1 (ICAM-1) (8–22) peptide or biotinylated 'scrambled' control peptide. Anti-biotin antibody was added, and colorimetric absorbance quantified the peptide-fibrinogen interaction. **(a) **Representative ICAM-1 (8–22) dose-response curve showing absorbance plateau at a fibrinogen concentration of approximately 100 μM. **(b) **Group mean absorbance data taken at the plateau fibrinogen concentration (100 μM) demonstrating a strong interaction between the ICAM-1 (8–22) peptide and fibrinogen compared with a small non-specific interaction between the scrambled peptide and fibrinogen. *p < 0.05 versus scrambled peptide.

By means of confocal microscopy, ICAM-1 was found to be present on the cell surface of TNF-α-activated cardiomyocytes. Co-immunostaining with fluorescently labeled fibrinogen demonstrated a high degree of colocalization (Figure 3a), supporting previous reports of interaction between these two molecules [17,21]. To confirm that fibrinogen specifically bound ICAM-1, we pre-treated isolated cardiomyocytes with either with an ICAM-1-blocking antibody or non-specific IgG. Compared with the IgG control group, cardiomyocytes treated with blocking antibody to ICAM-1 exhibited significantly lower adherence to fibrinogen-coated beads (Figure 3b). ICAM-1 is known to interact with fibrinogen via ICAM-1 peptides 8–22. To confirm this specific interaction between ICAM-1 and fibrinogen, we performed an ELISA binding assay using immobilized fibrinogen incubated with biotinylated ICAM-1 (8–22) peptide. In dose-finding experiments, as expected with receptor-ligand binding, there is a dose-response curve that reaches saturation at a fibrinogen concentration of 100 μM (Figure 4a). When group mean absorbance data are taken at this plateau fibrinogen concentration of 100 μM, there is a large increase in ICAM-1 (8–22) peptide binding with fibrinogen compared with scrambled peptide (Figure 4b). Thus, fibrinogen specifically binds to ICAM-1 through interaction with ICAM-1 peptides 8–22.

### Fibrinogen mediates decreased cardiomyocyte contractility via an ICAM-1-dependent mechanism

**Figure 5:**
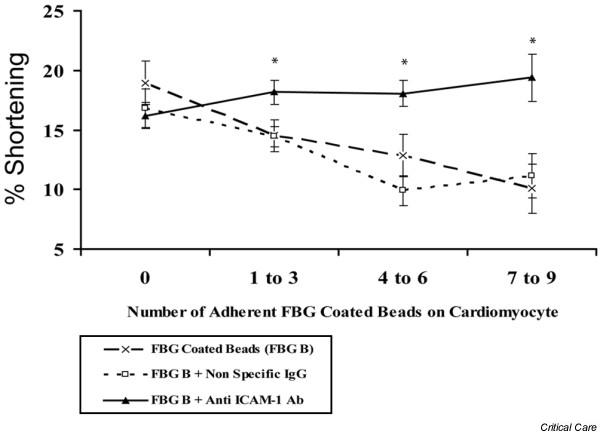
**Fibrinogen decreases cardiomyocyte fractional shortening via ICAM-1**. Cardiomyocytes were pre-treated with either a blocking anti-ICAM-1 antibody or isotype control antibody prior to the addition of fibrinogen-coated beads. Fractional shortening was then measured. Whereas pre-treatment with immunoglobulin G (IgG) isotype antibody was no different than fibrinogen alone, treatment with blocking anti-ICAM-1 antibody prevented the fibrinogen-induced decrease in fractional shortening. *p < 0.05 versus fibrinogen beads alone. Ab, antibody; FBG, fibrinogen; ICAM-1, intracellular adhesion molecule-1.

**Figure 6:**
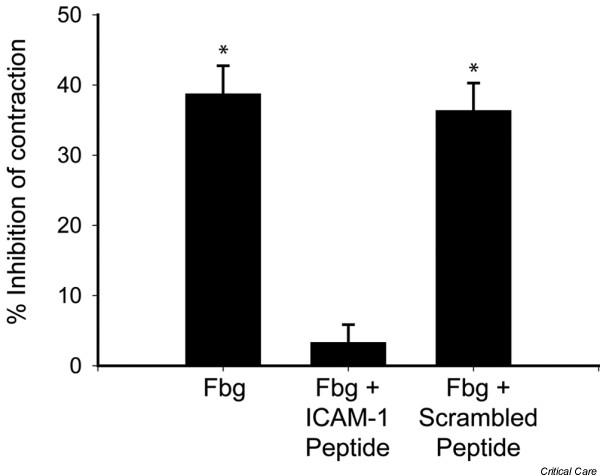
**ICAM-1 (8–22) mediates decreased contractility**. Before fibrinogen was added to activated cardiomyocytes, soluble intracellular adhesion molecule-1 (ICAM-1) (8–22) peptide, which binds fibrinogen at peptides 117–133, is added in excess to the fibrinogen. Activated cardiomyocytes incubated with fibrinogen alone demonstrate a 40% reduction in contractility. Pre-incubation of fibrinogen with the ICAM-1 (8–22) peptide results in competition between cardiomyocyte-expressed ICAM-1 and the ICAM-1 (8–22) peptide for the fibrinogen active site (117–133). Pre-incubation with the ICAM-1 (8–22) peptide abolishes the reduced contractility seen with fibrinogen alone, whereas pre-incubation with 'scrambled' ICAM-1 peptide had no effect. *p < 0.05 versus control. Fbg, fibrinogen.

We next examined whether ICAM-1 ligation by fibrinogen alters cardiomyocyte contractility. In one series of experiments, isolated cardiomyocytes were pre-treated with either ICAM-1-blocking antibody or isotype control antibody before adding fibrinogen-coated polystyrene beads. Whereas cardiomyocytes pre-treated with isotype control antibody demonstrated a dose-dependent decrease in contractility upon exposure to fibrinogen-coated beads, cardiomyocytes pre-treated with ICAM-1-blocking antibody demonstrated no reduction in contractility (Figure 5). To verify that this interaction is mediated specifically via the ICAM-1 (8–22) fibrinogen binding site, we performed a competitive assay in which a peptide containing the ICAM-1 (8–22) fibrinogen binding site was pre-incubated with soluble fibrinogen before addition of this mixture to activated isolated cardiomyocytes. Pre-incubation of the fibrinogen with excess ICAM-1 (8–22) peptide abolished the fibrinogen-mediated decrease in cardiomyocyte contractility, whereas pre-incubation of fibrinogen with a 'scrambled' peptide containing the same residues showed no such effect (Figure 6).

### Fibrinogen chain D mediates decreased cardiomyocyte contractility

**Figure 7:**
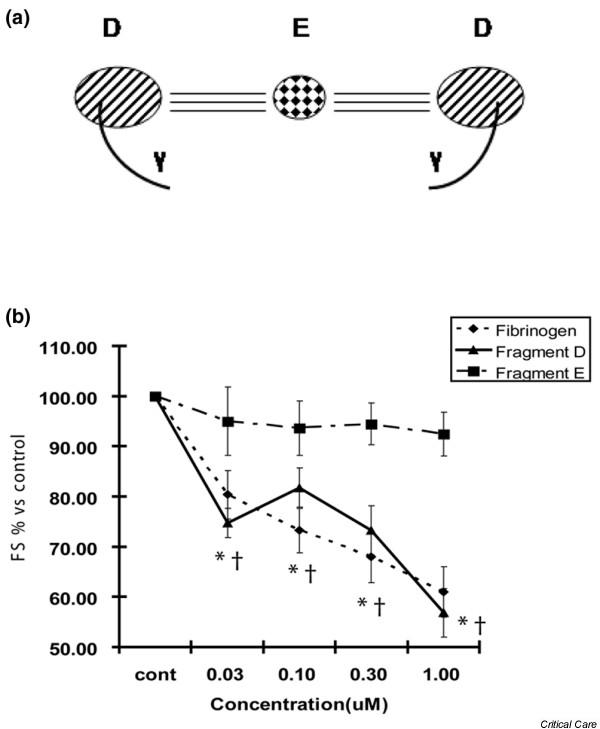
**Fibrinogen subunit D decreases contractility**. **(a) **Schematic diagram of the fibrinogen molecule, showing two D chains each containing a gamma chain linked to a central E chain. **(b) **Cardiomyocytes were incubated with whole fibrinogen as well as the major subunits D and E of fibrinogen. Whole fibrinogen and subunit D resulted in significant decreases in fractional shortening (FS), whereas subunit E had no significant effect. *,^†^*p *< 0.05 versus control.

Fibrinogen is composed of three major subunits, a central E chain linked to two D chains (Figure 7a). The smaller gamma chain is always found associated with the D chain and thus is not generally considered to be a distinct subunit. To determine whether the fibrinogen-mediated contractile dysfunction results from interaction of ICAM-1 with the intact whole molecule or whether a single chain contains the binding site, cardiomyocytes were incubated with whole fibrinogen, fibrinogen chain D, or fibrinogen chain E. There was a significant decrease in cardiomyocyte contractility following incubation with whole fibrinogen and fibrinogen chain D, but no effect was seen with fibrinogen chain E (Figure 7b).

### Amino acid sequence 117–133 of the fibrinogen gamma chain is the active site, and the crosslinked gamma chains of D-dimer retain the ability to interact with ICAM-1

**Figure 8:**
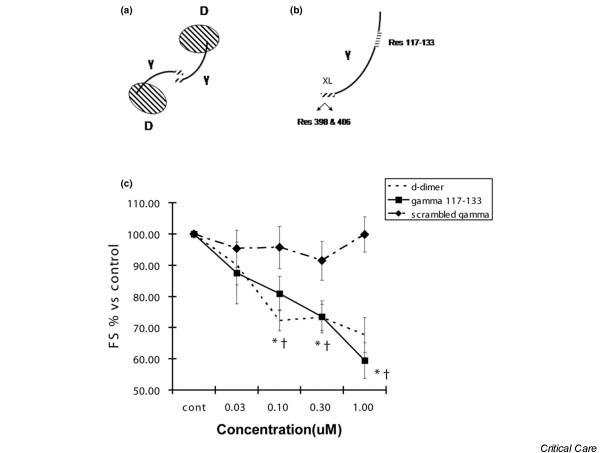
**Fibrinogen gamma chain and D-dimer decrease contractility**. **(a) **Schematic diagram of D-dimer with two D subunits linked in part via interaction of the gamma chains. For simplicity, we have not shown the areas on the D chain itself which participate in dimerization. **(b) **Expanded diagram of the gamma subunit showing the crosslinking (XL) site of the C-terminal regions which results in amine donor lysine 406 of one gamma chain and a glutamine acceptor at residue 398 or 399. The intracellular adhesion molecule-1 (ICAM-1) binding site is shown as residue 117–133, far removed from the XL site. **(c) **Cardiomyocytes were incubated with a peptide with the sequence 117–133, D-dimer, or scrambled peptide. D-dimer and the gamma (117–133) peptide resulted in significant decreases in fractional shortening (FS), whereas scrambled peptide had no significant effect. *,^†^*p *< 0.05 versus control.

We next determined whether the ICAM-1 binding gamma chain site 117–133 [17] of the D chain (Figure 8a) was responsible for the observed contractile dysfunction. Furthermore, as dimerization of fibrinogen chain D (commonly known as D-dimer) is accomplished in part via crosslinking the XL sites of the gamma chain (Figure 8a,b) [27], we tested whether dimerization resulted in attenuation of the ICAM-1-mediated effect. The gamma peptide 117–133 resulted in a significant reduction in fractional shortening compared with control, whereas scrambled peptide had no effect (Figure 8c). Incubation with D-dimer also resulted in a significant reduction in fractional shortening (Figure 8c), demonstrating that the functional site 117–133 remains active despite crosslinking of gamma chains.

## Discussion

In this study, we propose a novel mechanism linking two phenomena that occur as a result of inflammation: dysregulation of the coagulation cascade and myocardial dysfunction. The key finding reported is that amino acid sequence 117–133 of the fibrinogen gamma chain decreases cardiomyocyte contractility through binding ICAM-1. Of great interest to clinicians is that D-dimer, a product of fibrinogen polymerization and subsequent digestion which includes 117–133 of the gamma chain, in addition to its important role in diagnosis of thromboembolism, can decrease cardiomyocyte contractility.

It has long been recognized that seemingly disparate causes of local or systemic inflammation, such as ischemia reperfusion, inflammatory cardiomyopathy, orthotopic heart transplant rejection, or sepsis [1-3], all culminate in myocardial dysfunction. While each disorder undoubtedly poses unique challenges to the maintenance of myocardial homeostasis, there could be a factor that is common to all. Endothelial damage with subsequent capillary leakage represents a final common pathway of inflammatory disorders [28]. Increased permeability of the endothelium leads to a shift of circulating elements from the plasma into the organs. Should this fluid flux contain circulating substances capable of depressing myocardial contractility, this may be the link between myocardial dysfunction and inflammatory states. This depressant not only must reach the cardiac myocytes but must have a receptor capable of mediating changes in contractility. We have previously shown that ICAM-1 expressed on cardiomyocytes is induced by inflammatory mediators and, upon activation, is capable of decreasing cardiomyocyte contractility [9,13].

Any circulating ICAM-1 ligands could be candidates for causing myocardial dysfunction provided that they permeate the heart. CD11a/CD18 (LFA-1) and Cd11b/CD18 (Mac-1) expressed on the surface of polymorphonuclear leukocytes are ICAM-1 ligands capable of reducing myocyte contractility *in vitro *[11] and have been proposed to be the link between inflammation and cardiac dysfunction. However, we and others have noted a striking paucity of intramyocardial leukocytes in whole animal models of inflammation [12,13]. Fibrinogen, as well as its related protein fragments D-dimer and other FDPs, represents potential ICAM-1 binding myocardial depressant substances. Through both human epidemiologic data and basic science, fibrinogen and FDPs satisfy two major criteria for causality. Clinically, not only are fibrinogen and D-dimer markedly increased in inflammatory disorders, but their levels are inversely correlated with favorable outcome [4-8]. As for biologic plausibility, the amino acid sequence 117–133 of fibrinogen gamma chain (fg-γ-117–133) is capable of binding the amino acid sequence 8–22 (ICAM-1-8–22) within the first Ig domain of ICAM-1 [17]. While the fibrinogen-ICAM-1 interaction facilitates adhesion of leukocytes to endothelial cells [18,19], leukocyte transmigration [20], and promotion of endothelial cell survival [21], there is no information regarding its role in cardiac physiology.

In this study, we show for the first time that fibrinogen is capable of mediating a reduction in cardiomyocyte contractility thorough activation of ICAM-1. It is important to note that significant reduction in fractional shortening was achieved at a fibrinogen concentration of 0.2 mg/mL, approximately one order of magnitude less than its physiological concentration in plasma [27]. Fibrinogen is a large 340-kDa plasma glycoprotein and, as such, would be expected to have limited tissue penetration compared with smaller plasma proteins. Despite its large size, however, we showed a significant increase in perivascular fibrinogen deposition in an *in vivo *model of systemic inflammation. FDPs, notably fragment D (100 kDa) or D-dimer at roughly double that size [27], could potentially infiltrate deeper into the myocardium and exert their depressant effect in areas that fibrinogen could not access. Importantly, not only did we find that systemic injury increased intracardiac fibrinogen, there was a dramatic increase in ICAM-1 expression. Thus, our animal models of disease provide *in vivo *evidence to support the hypothesis that fibrinogen and FDPs might be the circulating myocardial depressant factors.

Determining whether the previously identified interaction between fibrinogen and ICAM-1 [17-21] is responsible for alterations in cardiac physiology is fundamental information required both to understand the mechanism and to design potential therapeutics. Fibrinogen consists of two major chains, E and D, as illustrated in Figure 7a. The gamma chain is a subunit of chain D, as shown in Figures 7a and 8a, and contains a crosslinking (XL) site through which two D chains dimerize. The putative active site of fibrinogen is 117–133 on the gamma chain [17] and, though remote from the XL site as shown in Figure 8b, might be altered or allosterically interfered with upon dimerization. We show that fibrinogen chain D causes cardiomyocyte contractile dysfunction whereas chain E had no biologic effect. This agrees with findings by other investigators that it is the D chain responsible for ICAM-1-mediated vasoconstriction [29]. Furthermore, we went on to show that previously identified [17] site 117–133 of the fibrinogen gamma chain was responsible for the ICAM-1-mediated physiologic effects. As the gamma chain is linked to the D chain, it is important to consider it in the context of polymerized D chains. Despite its crosslinked gamma chains, D-dimer also caused significant reductions in contractility. Although the specificity of interaction of D-dimer with fibrinogen was not tested through antibody blockade or competing peptide, we believe that, as it is composed of two D subunits, the mechanism of action is nearly certainly analogous to the individual D chain. This last finding is particularly exciting given that, while long touted as a biomarker for both the presence and prognosis of inflammatory disease [4-8], D-dimer has been perceived, to date, as a disease marker with no intrinsic biologic effect. Not only do we now know that vascular tone can be altered by the fibrinogen D chain's activation of ICAM-1 [29], but here we demonstrate that the key contractile function of cardiac muscle cells is impaired via the same mechanism.

## Conclusion

Site 117–133 of the fibrinogen gamma chain is able to depress cardiomyocyte contractility through binding ICAM-1. The implication of the reported mechanism extends beyond the realm of our models of inflammation to other pathologies characterized by inflammation and heart failure, such as post-ischemia reperfusion injury, inflammatory cardiomyopathy, and orthotopic heart transplant rejection.

## Key messages

• Intracardiac intracellular adhesion molecule-1 (ICAM-1) and fibrinogen are increased as a result of systemic inflammation.

• Amino acid sequence 117–133 of the fibrinogen gamma chain is responsible for binding ICAM-1, functionally decreasing cardiomyocyte contractility.

• D-dimer contains the fibrinogen gamma chain and also decreases cardiomyocyte contractility.

## Abbreviations

BSA = bovine serum albumin; EDV = end diastolic volume; E_es _= end-systolic elastance; EF = ejection fraction; ELISA = enzyme-linked immunosorbent assay; E_max _= maximal end-systolic elastance; FDP = fibrinogen degradation product; FITC = fluorescein isothiocyanate; ICAM-1 = intracellular adhesion molecule-1; Ig = immunoglobulin; LAD = left anterior descending; LPS = lipopolysaccharide; PBS = phosphate-buffered saline; TNF-α = tumor necrosis factor-alpha; vWF = von Willebrand factor; XL = crosslinking.

## Competing interests

The authors declare that they have no competing interests.

## Authors' contributions

JHB drafted the manuscript. EHC performed the initial contractility measurements. EYD helped design the contractility experiments. CT performed the peptide experiments. RMB performed immunohistochemistry. GH designed the peptides. YW performed the animal work. KRW conceived of the study, participated in its design and coordination, and helped to draft the manuscript. All authors read and approved the final manuscript.
